# 4-D PET joint image reconstruction/non-rigid motion estimation with limited MRI prior information

**DOI:** 10.1186/2197-7364-1-S1-A27

**Published:** 2014-07-29

**Authors:** Alexandre Bousse, Jieqing Jiao, Kjell Erlandsson, Luis Pizarro, Kris Thielemans, Dave Atkinson, Sébastien Ourselin, Simon Arridge, Brian Hutton

**Affiliations:** Institute of Nuclear Medicine–UCL, University College London, London, NW1 2BU UK; Centre for Medical Image Computing, University College London, London, WC1E 6BT UK; Centre for Medical Radiation Physics at the University of Wollongong, NSW, Australia

Motion compensated gated PET image reconstruction methods include joint-reconstruction (JR) and indirect reconstruction (IR) with pre-estimated motion from MRI (MRI-IR). JR suffers from poor PET data quality whereas MRI-IR requires high-quality MRI volumes at each gate. We propose a penalised maximum-likelihood approach combining JR and MRI-IR. Our method is referred to as *minimal MRI prior* JR (MP-JR).

The *M* gates data are stored in ***g*** = [***g***_1_; …; ***g***_*M*_] where ***g***_*m*_ is the measurement vector at gate *m*. Each ***g***_*m*_ is a Poisson distributed vector of parameter  where ***P*** is the projector, *W*(***α***_*m*_) is the *m*-th motion of parameter ***α***_*m*_, ***r***_*m*_ is the *m*-th average random/scatter vector and ***f*** is the activity at *m* = 1. JR is achieved with (1).1

MRI-IR is achieved by solving (2)2

MP-JR is achieved with (3).3

The first term accounts for PET data, whereas the second term accounts for MRI motion information from subset *S*. The last term controls temporal smoothness.

We tested each method on 9 PET FDG volumes generated from a real dynamic MRI sequence. Tumours were added to the activity distribution (invisible in the MRI). The gates subset *S* for MP-JR contains the reference gate, end-inspiration and end-expiration. Reconstruction profiles 1 show that MRI-IR improves edges visible in the MRI but degrades the tumours. On the contrary, JR performs well on tumours, but the edges are poorly reconstructed. MP-JR appears to perform well on both organ edges and tumours.

**Figure 1:**
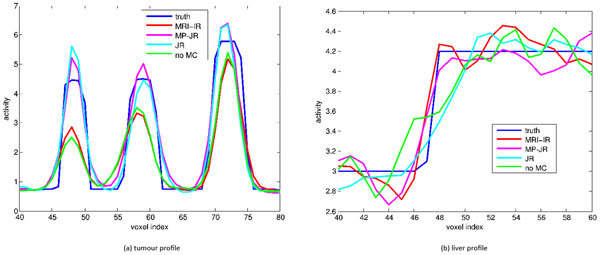
Reconstruction profiles: (a) across tumours; (b) across liver.

MP-JR seems to perform well where both JR and MRI-IR under-perform. This is due to the fact that MP-JR relies on both MRI and PET data. In addition, results tend to show that with temporal smoothing on B-spline parameters, a subset of MRI volumes provides sufficient information.

